# Effects of Syringic Acid on Apoptosis, Inflammation, and AKT/mTOR Signaling Pathway in Gastric Cancer Cells

**DOI:** 10.3389/fnut.2021.788929

**Published:** 2021-12-14

**Authors:** Jinjin Pei, Periyannan Velu, Mohsen Zareian, Zili Feng, Annamalai Vijayalakshmi

**Affiliations:** ^1^College of Bioscience and Bioengineering, Qinba State Key Laboratory of Biological Resources and Ecological Environment, QinLing-Bashan Mountains Bioresources Comprehensive Development C. I. C, Shaanxi Province Key Laboratory of Bio-Resources, Shaanxi University of Technology, Hanzhong, China; ^2^Department of Biology and Biological Engineering, Chalmers University of Technology, Göteborg, Sweden

**Keywords:** plant-derived phytochemicals, therapeutic compounds, phytochemical therapy, cancer cell lines, apoptosis, drug resistance, molecular mechanism, antitumor activity

## Abstract

Gastric cancer is one of the most common cancer and deadly disease worldwide. Despite substantial advances made in the treatment of gastric cancer, existing therapies still encounter bottlenecks. Chemotherapy, for instance, could lead to serious side effects, high drug resistance and treatment failure. Phytochemical-derived compounds from plants offer novel strategies as potent drug molecules in cancer therapy. Given the low toxicity and higher tolerance rate of naturally occurring compounds, the present study evaluated the effects of syringic acid on cytotoxicity, oxidative stress, mitochondrial membrane potential, apoptosis, and inflammatory responses in gastric cancer cell line (AGS). AGS cells were treated with various concentrations (5–40 μg/mL) of syringic acid for 24 h, after which cytotoxicity was analyzed. Reactive Oxygen Species (ROS), antioxidant enzyme activities, mitochondrial membrane potential (MMP, Δ*ψ*_m_), cell morphologies, the expression of apoptotic markers and protein expression patterns were also investigated. Results indicated that syringic acid-treated cells developed anti-cancer activities by losing MMP, cell viability, and enhancing intracellular ROS. Syringic acid selectively developed apoptosis in a dose-dependent manner via enhanced regulation of caspase-3, caspase-9 and Poly ADP-ribose Polymerase (PARP) whereas decreasing the expression levels of p53 and BCL-2. Syringic acid also lowered activities of superoxide dismutase (SOD), catalase (CAT) and glutathione peroxidase (GSH-Px) whereas Thio Barbituric Acid Reactive Substances (TBARS) increased. Syringic acid suppressed gastric cancer cell proliferation, inflammation, and induced apoptosis by upregulating mTOR via AKT signaling pathway. The study suggests syringic acid may constitute a promising chemotherapeutic candidate for gastric cancer treatment. Our study is the first report on the anti-cancer effects of syringic acid against gastric cancer cells via apoptosis, inhibition of inflammation, and the suppression of the mTOR/AKT signaling pathway.

## Introduction

Gastric cancer is ranked as the 5^th^ most common cancer and the 3^rd^ most deadly disease worldwide, with high mortality rates in developing countries (1, 2). The risk of gastric cancer can be exacerbated by smoking, inadequate diet, obesity, environmental factors, age, infections caused by *Helicobacter pylori* and genetics (3). Recently, multiple therapies such as surgical excision with additional adjuvant chemoradiation or chemotherapy were suggested to increase the survival time of gastric cancer patients up to 5 years (1, 4, 5). However, chemotherapy could lead to serious side effects, high drug resistance, and treatment failure (6). Interest has particularly raised toward phytochemicals and naturally occurring compounds present in dietary natural products for gastric cancer treatment purposes - thanks to low toxicity and higher tolerance rate of plant-derived phytochemicals (7–9).

Phytochemicals derived from herbs (e.g., celastrol, curcumin, matrine, ginsenoside, and silibinin) have demonstrated stable efficacy and minimal toxicity toward gastric carcinoma patients (10). Syringic acid (4-hydroxy-3, 5-dimethoxybenzoic acid) is a naturally occurring compound which can be found in cereal grains, shiitake mushroom, olives, spices, pumpkin, grapes, honey, the leaves of *Morus nigra, Radix Isatidis, Herba dendrobii* and *Alpinia calcarata Roscoe* (11). Syringic acid possesses various physiological functions such as anti-inflammatory, anti-mitogenic, anti-oxidant, anti-cancer, anti-diabetic and hepatoprotective properties (11).

Studies have shown syringic acid can modulate proteins, transcriptional factors, growth factors and signaling molecules, particularly in various cancer cells (11, 12). Syringic acid can also alter soil rhizosphere microbiota, therefore plays a role in the communication between plant – microbes (13). Majority of syringic acid applications in industry has been focused on foods and cosmetics, water treatments, bioremediation, photocatalytic ozonation and laccase-based detoxification (11).

Given the urge to develop new anti-cancer drugs, plant-based bioactive phytochemicals offer high efficiency and low toxicity characteristics that could ultimately be used for the clinical treatment of gastric cancer patients. Therefore, the present study investigated whether syringic acid could exhibit any effects against gastric cancer cells and to provide an insight into the molecular pathways. The toxicity effect of syringic acid is not however investigated in the present work.

## Materials and Methods

### Chemicals and Reagents

Syringic acid, trypsin-EDTA solution, dichloro-dihydro-fluorescein diacetate (DCFH-DA), 4,6-diamidino-2-phenylindole (DAPI), propidium iodide (PI), Nitro Blue Tetrazolium (NBT), Sodium Dodecyl Sulfate (SDS), malondialdehyde bis(dimethyl acetal) and sodium nitrate were procured from Sigma (Steinheim, Germany). Dulbecco's modified eagle medium (DMEM), penicillin, streptomycin, β-actin, Phosphate-Buffered Saline (PBS), 3-(4,5-Di-2-yl)-2,5-ditetrazolium bromide (MTT), fetal bovine serum (FBS), Rhodamine123 (Rh-123), xanthine, catalase, glutathione reductase, Nicotinamide Adenine Dinucleotide Phosphate (NADPH) were acquired from Gibco (Suzhou, China).

### Maintenance of Cell Culture

Gastric cancer cell lines (AGS) were secured from Peking Union Cell Resource Center (Beijing, China) and cultured in a DMEM medium containing 10% FBS and 1% antibiotic solution. Cells were sustained at 37°C under 5% CO_2_, and the nutrient medium was replaced after 2–3 days. Cell passage was performed using the trypsin-EDTA solution to obtain a 75–80% confluency.

### Cell Viability Assay

Cytotoxicity effects of syringic acid were assessed by MTT assay as previously described (14). In 96-well plates, 1 × 10^4^ cells per each well were first seeded, after 24 h, treated with syringic acid (5, 10, 15, 20, 25, 30, 35, and 40 μg/mL) and incubated for 24 h. The MTT solution (10 μL) was added and incubated for 4 h in a CO_2_ incubator, after which absorbance was measured at 540 nm by a microplate reader (BioTek Instruments, Colmar, France). Cell viability was defined as percentage to untreated cells and calculated as follows: Cell viability % = (Abs _treatedcells_ /Abs _untreatedcells_) × 100.

### DCFH-DA Staining

Dichloro-dihydro-fluorescein diacetate (DCFH-DA) staining was used to detect ROS in cells. In brief, cells were first seeded, allowed to achieve cell adhesion for 24 h, after which exposed to 25 and 30 μg/mL syringic acid. A 15 μg DCFH-DA stain was introduced to the cells and incubated in the dark at 37°C for 30 min, centrifuged and washed 3 times using ice-cold PBS to remove excess DCFH-DA staining and measured by spectrofluorometer emission filters at 488/530 nm. Ultimately, fluorescence microscopy images of the control and treated cells were measured at 450/490 nm.

### Acridine Orange (AO) and Ethidium Bromide (EB) Staining

Apoptotic morphological changes were determined by acridine orange/ethidium bromide (AO/EB) dual staining as previously described (15). In brief, AGS cells (1 × 10^4^ cells per each well) were first seeded in 96-well plates, after 24 h cells were treated with 25 and 30 μg/mL syringic acid and incubated at 37°C for 2 h. Cells were washed with PBS and stained with AO/EB dual stains (1:1 ratio). The control and syringic acid-treated cells were stained by AO (100 μg/mL) and EB (100 μg/mL) for 5 min, after which, cell morphologies were evaluated using a fluorescent microscope.

### DAPI Staining and Mitochondrial Membrane Potential

The diamidino-2-phenylindole (DAPI) staining was carried out as previously described (16), and Mitochondrial Membrane Potential (MMP) was also measured by Rh-123 (17). Cells were first added to 6-well plates for adherence and incubated at 37°C for 24 h in an incubator containing 5% CO_2_. Cells were treated with 25 and 30 μg/mL concentrations of syringic acid for 24 h, after which, washed twice with PBS, stained with DAPI and incubated for 20 min. Subsequently, DAPI-stained cells were treated with Rh-123 stain at 37°C for 30 min, washed twice with PBS to remove the surplus stains, and analyzed for changes in Δ*ψ*_m_ using fluorescent microscopy with 40× magnification.

### Propidium Iodide Staining

To analyse DNA fragmentation in apoptosis, the Propidium iodide (PI) staining assay was carried out as previously described (18). After cell incubation, 25 and 30 μg/mL syringic acid were added to the cells for 24 h. Cells were washed twice with PBS and stained by PI (10 μg/mL) for 5 min after which analyzed by fluorescence microscopy.

### Antioxidant Assays

AGS cells (1 × 10^4^ cells per each well) were first seeded, after 24 h, cells were treated with 25 and 30 μg/mL syringic acid and incubated at 37°C for 2 h. Cells were harvested for superoxide dismutase (SOD), catalase (CAT), and glutathione peroxidase (GSH-Px) and thiobarbituric acid reactive substances (TBARS) assays as follows: For SOD assay the xanthine-xanthine oxidase system to reduce Nitro Blue Tetrazolium (NBT) was performed as previously described (19). The reaction mixture contained 1 mM EDTA, 50 mM potassium phosphate, 100 μM xanthine, catalase (100 U/mL), 56 μM NBT, 0.02% Triton X-100, 006% BSA and cellular lysate (0.125 mg protein) at pH 7.8 and 37°C. The absorbance was recorded for 5 min at 560 nm for monitoring NBT reduction. Upon a steady state absorbance change, the reaction was started by adding xanthine oxidase (XO, 8 mU/mL). The highest NBT reduction (100%) was obtained by replacing cellular sample with distilled water in the reaction tube. Total protein concentration was measured by the method of Bradford (20) and bovine serum albumin was used as standard. The amount of protein which inhibited 50% NBT reduction was defined as one unit of SOD activity. Results were expressed as 50% NBT reduction/min/mg protein.

The TBARS assay was carried out as previously described (21). For malondialdehyde standard, a 200 μM MDA bis(dimethyl acetal) solution was prepared fresh every time the TBARS assay was performed. In a glass tube, 200 μL sodium dodecyl sulfate (SDS 8% *w/v*) was added to 100 μL sample and gently whirled, after which 1.5 mL sodium acetate buffer (3.5 M, pH 4) and 1.5 mL thiobarbituric acid solution (aqueous 0.8%, pH 4) was added. The final volume was brought to 4 mL by adding distilled water. The reaction mixture was vortexed and heated in boiling water for 45 min, after which, cooled and centrifuged at 6000 rpm for 10 min. The absorbance of supernatants was read at 532 nm using a spectrophotometer (Novaspec II VisibleSpectro, Pharmacia Biotech, Uppsala, Sweden). MDA level was reported as nmol MDA per mg protein.

The activity of GSH-Px was determined as previously described (22). In brief, to each sample was added 2.68 mL phosphate buffer (0.05 M, pH 7.0), 0.1 mL ethylenediaminetetraacetic acid (EDTA, 0.005 M), 0.01 mL glutathione reductase, 0.10 mL NADPH (0.0084 M), 0.01 mL sodium nitrate (1.125 M), and 0.1 mL reduced glutathione (GSH, 0.15 M). A 0.1 mL 0.0022 M hydrogen peroxide was added to the mixture to initiate the enzymatic reaction. The conversion of NADPH to oxidized NADP^+^ was recorded by the changes in the absorbance at 340 nm between 2–4 min once the enzymatic reaction was initiated. An equal volume of cell lysis buffer was used as a control. One unit of GSH-Px enzyme activity was defined as 1 mM NADPH as converted to mM NADP^+^ utilized per mg protein per min.

The activity of CAT was determined as previously described (23). Briefly, to 10 μL supernatant was added 50 μL H_2_O_2_ (0.036% *w/w*) and incubated at 37°C for 1 min, after which the reaction was ceased by addition of sulfuric acid 5N and potassium permanganate solution 0.005 N. The reaction yielded a pink color and measured at 490 nm. The difference in absorbance per unit indicated catalase activity and data were expressed as μmol H_2_O_2_ utilized/min/mg protein. All experiments were performed in triplicates and results were shown as mean ± standard deviation.

### Real-Time Polymerase Chain Reaction (RT-PCR)

Total RNA was isolated using the Rneasy Mini Kit (Qiagen, Hilden, German) as per the manufacturer's protocol. cDNAs were synthesized from 1 μg of total RNA using ImProm-II Reverse Transcriptase (Promega, Madison, USA). The set of primers used in the PCR reactions are listed in Table 1. A 20 μL sample was used in the amplification reaction at 95°C for 5 min, followed by 28 cycles at 95°C for 30 s, at 54°C for 45 s, at 72°C for 30 s, and ultimately 10 min at 72°C. To validate the effect of cDNA synthesis from modified cells, β-actin was used as a control. PCR products were investigated on 1.2% Seakem agarose gels.

**Table 1:** List of primer sets used in the study.

**Primer sets**	**Sense**	**Anti-sense**
Nuclear factor-kappa B (NF-κB)	5′-ATGGACGATCTGTTTCCCCT-3′	5′- CGGTTTACTCGGCAGATCTT-3′
Cyclooxygenase-2 (COX-2)	5'-TGGGCCATGGAGTGGACTTA-3'	5'-ATGAGCCTGCTGGTTTGGAA-3'
Tumor necrosis factor-α (TNF-α)	5′-TCTGGGCAGGTCTACTTTGG-3′	5′-TCTTCTCAAGTCCTGCAGCA-3′
Interleukin-6 (IL-6)	5′-AAACAACCTGAACCTTCCAAAGA-3′	5′-GCAAGTCTCCTCATTGAATCCA-3′
β-actin	5'-AACCGCGAGAAGATGACCCAGATCATGTTT-3'	5'-AGCAGCCGTGGCCATCTCTTGCTCGAAGTC-3'

### Western Blotting

For Western blotting, syringic acid-treated cells were added to the protein lysis buffer and incubated at 4°C for 30 min, after which centrifuged with 14,000 rpm at 4°C for 30 min. The protein concentration in the supernatant was measured by the Bradford method. From the cell extracts, 25 and 30 μg/mL were subjected to SDS-PAGE based upon mass or volume from 8–15% and relocated to polyvinylidene fluoride (PVDF, Bio-Rad, CA, USA). The 5% skim milk was added in TBST buffer (153 mM NaCl, 10 mM Tris-HCl, 0.05% Tween 20, pH 7.5) at 20°C and incubated for 1 h. The PVDF membranes were incubated with primary antibody (p53, B-cell lymphoma 2 (BCL-2), Bad, caspase-3, caspase-9, Poly (ADP-ribose) polymerase (PARP), phosphorylated Akt (p-Akt), protein kinase B (AKT), phosphorylated mTOR (p-mTOR), mammalian target of rapamycin (mTOR), and β-actin at 4°C overnight. The PVDF membranes were washed thrice by TBST and incubated with secondary antibody (HRP conjugated) at 25°C for 2 h. The PVDF membranes were again washed thrice by TBST buffer. Enhanced chemiluminescence (ECL) detection agent (Thermo Fisher Scientific, Waltham, MA, USA) was employed to develop blots.

### Statistical Analyses

Data were expressed as mean ± standard deviation in triplicate equivalent experiments. The statistical analyses were performed using one-way ANOVA by SPSS (Ver. 25.0, SPSS Inc., Chicago, IL, USA) and α at 95% was defined as a statistically significant difference.

## Results

### Cell Viability

**Figure 1:**
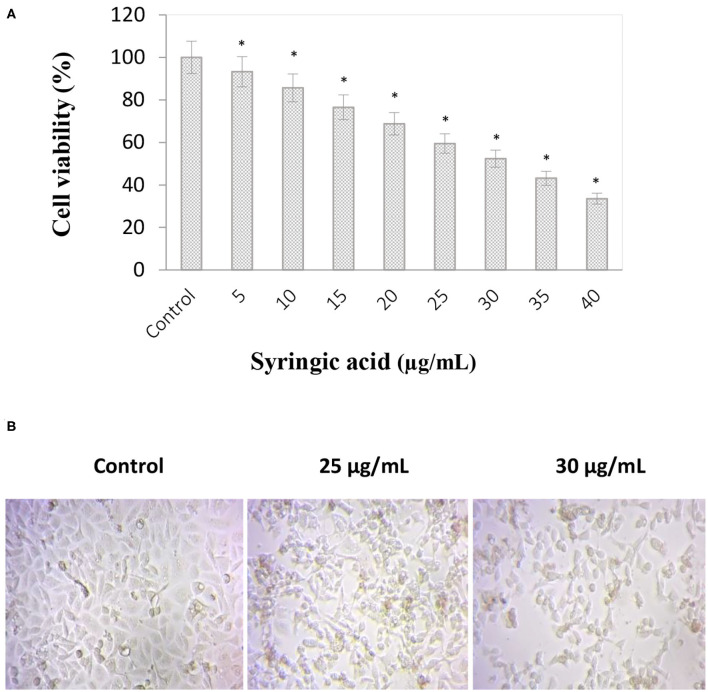
Anti-proliferative effects of syringic acid on gastric cancer cells. **(A)** Cells were treated with various concentrations of syringic acid (5–40 μg/mL) for 24 h, and data were expressed as a percentage to the control to demonstrate cell cytotoxicity ratio. **(B)** Morphological changes in cancer cells upon a 24 h-treatment with 25 and 30 μg/mL syringic acid. The 50% cell death occurred in the concentration ranges between 25–30 μg/mL of syringic acid. Values are the mean ± standard deviation of three independent experiments.

Syringic acid cytotoxicity effect at varying concentrations (5–40 μg/mL) against gastric cancer cells is represented in Figure 1A. The inhibition concentration (IC_50_) of syringic acid was found to be at 30 μg/mL and 50% of cell death occurred in the concentration ranges between 25 and 30 μg/mL. Therefore 25 and 30 μg/mL doses of syringic acid were selected for further investigations. A significant loss of morphological changes was also observed at 25 μg/mL syringic acid treatments, and the increase in syringic acid dose to 30 μg/mL resulted in further morphological alterations (Figure 1B).

### Morphological Changes, MMP (Δ*ψ*^m^), ROS Production and Antioxidant Status

**Figure 2:**
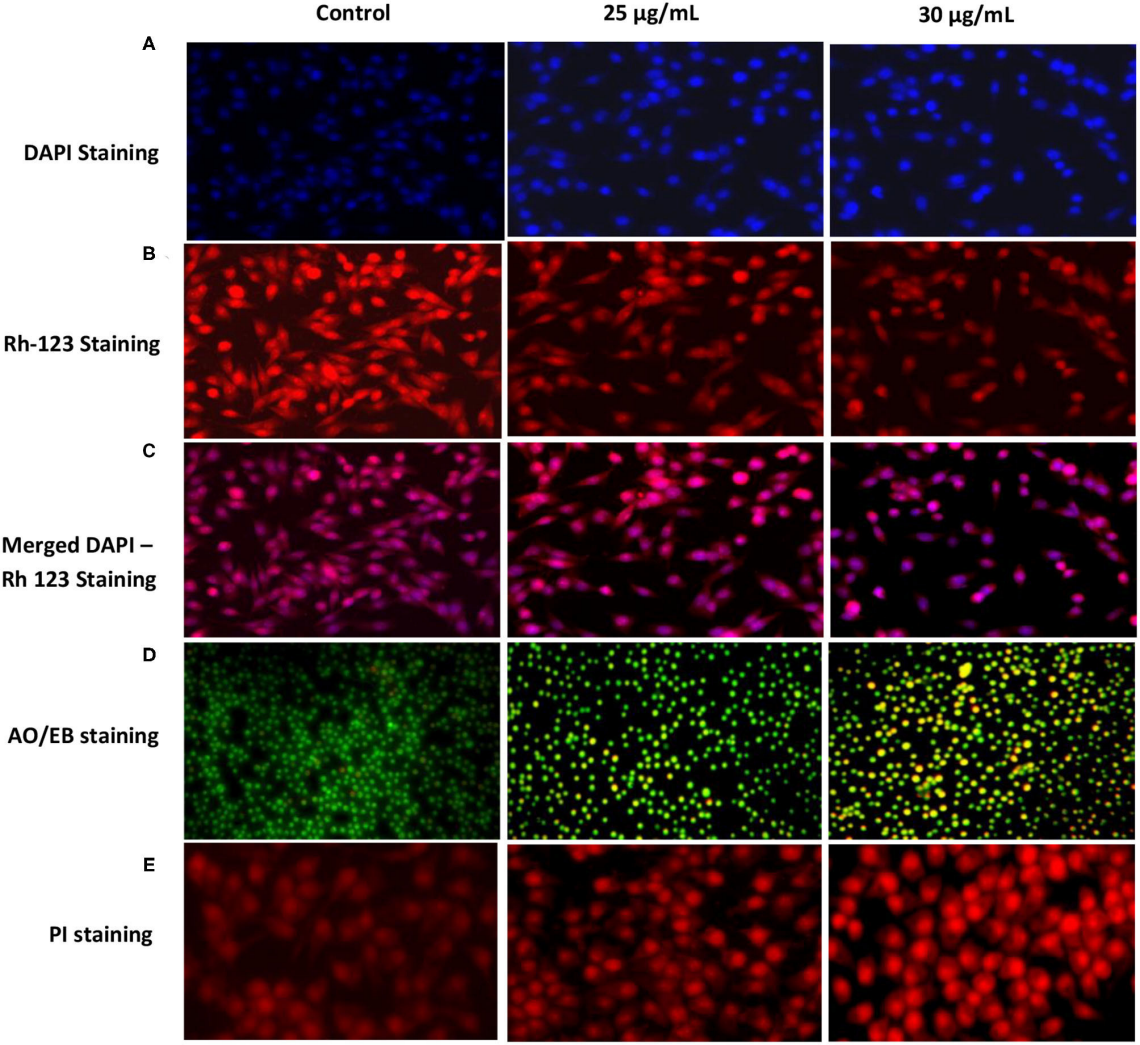
Effects of syringic acidin gastric cancer cells on mitochondrial membrane potential by DAPI staining **(A)**, Rh-123 staining **(B)**, and merged DAPI - Rh-123 **(C)**. Nuclear morphological changes were investigated by AO/EB and PI stainings **(D,E)**. Early apoptotic cells indicate green fluorescence; apoptotic cells indicate yellow fluorescence; late apoptotic cells indicate orange fluorescence. Cells were incubated for 24 h, and images depict 40× magnifications using a fluorescence microscope.

Images of DAPI-stained cells with nuclear condensation and fragmentation indicated a significant increase in apoptotic cells upon treatment with 30 μg/mL syringic acid than the control (Figure 2A). The mitochondrial membrane potential (Δ*ψ*_m_) of cells treated with syringic acid (25 and 30 μg/mL) is also illustrated (Figure 2B). The fluorescence dye red colouration highlights the accumulation of Rh-123 whereas the decrease in Rh-123 accumulation can be observed as yellow greenish (Figures 2B,C). The finding therefore indicates that Δ*ψ*_m_ decreased in syringic acid-treated cells which consequently led to mitochondrial-mediated apoptosis. Instead, substantial apoptosis-mediated morphological changes (i.e., apoptotic body formation and chromatin condensation) were observed. The apoptotic morphological changes demonstrated by dual staining AO/EB indicated red fluorescence stains of EB, which entered nuclei of apoptotic cells (Figure 2D). In contrast, a green dye of AO was generated only by un-damaged cells. Control cells showed a bright green fluorescence nucleus, which indicates live cells (Figure 2D). Cells also showed orange colouration, indicating untimely apoptosis, whereas red-stained cells indicate DNA damaged and delayed apoptosis (Figure 2D). The PI staining also showed apoptotic and necrotic changes suggesting that 30 μg/mL syringic acid induced apoptotic morphological damage in gastric cancer cells compared to the control (Figure 2E).

**Figure 3:**
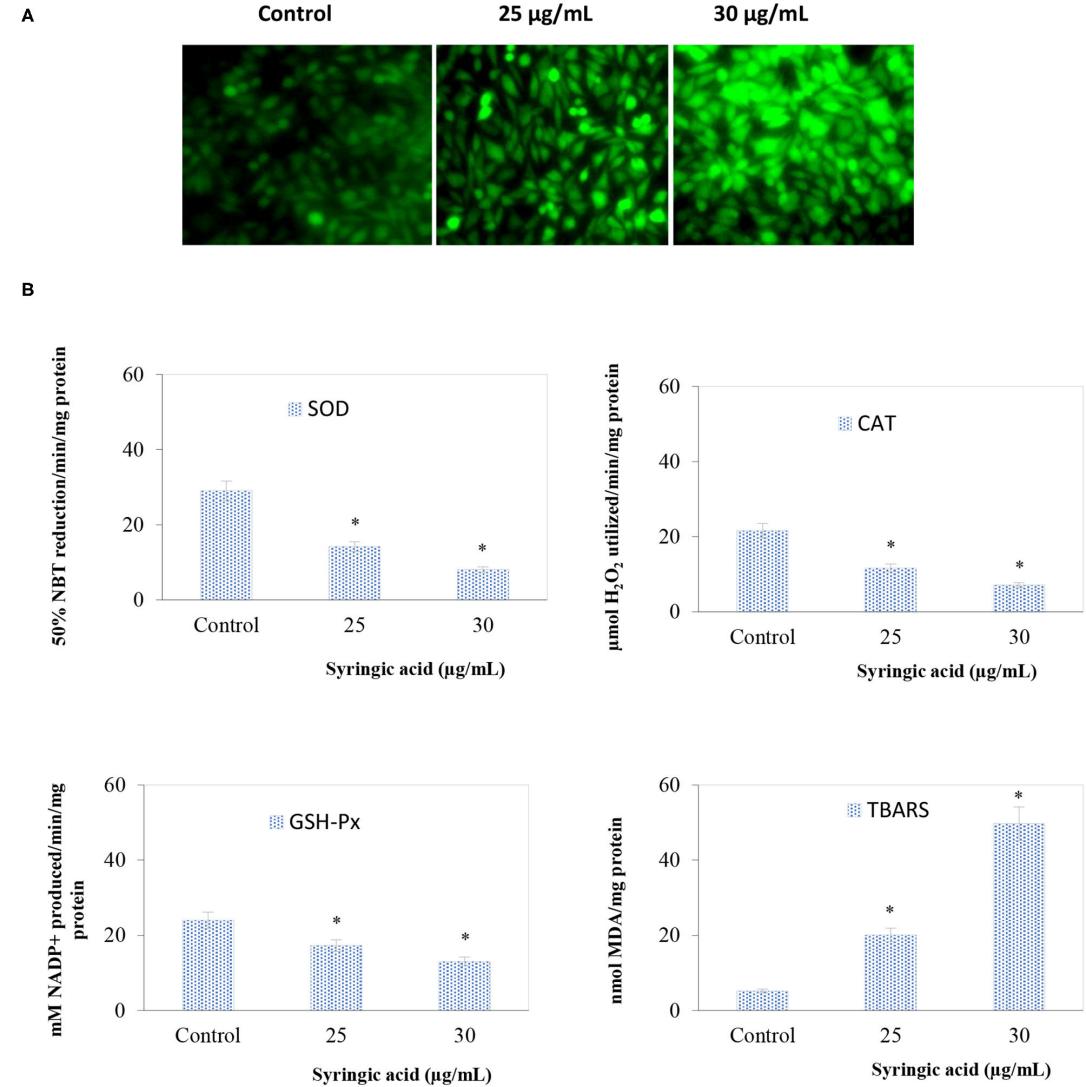
**(A)** Syringic acid induces intracellular ROS generation in AGS gastric cancer cells by DCFH-DA staining in which DCF fluorescence increased in a dose-depended manner of syringic acid (25 and 30 μg/mL) **(B)** Effects of syringic acid on antioxidant and lipid peroxidation enzymes actions. Cells treated with 25 and 30 μg/mL syringic acid for 24 h show a reduction in SOD, CAT, and GSH-Px, whereas an increase in TBARS. Values are the mean ± standard deviation of 3 independent experiments.

The ROS status in samples treated with 25 and 30 μg/mL syringic acid significantly (*P* < 0.05) increased (Figure 3A) and the oxidative stress in the antioxidant enzymes (GSH-Px, CAT, SOD) declined indicating the ROS generation process (Figure 3B). Such observation is because of two methoxy groups attached to the aromatic ring of syringic acid molecule at positions 3 and 5 (11, 24).

### Expression of Molecular Markers, Pro-inflammatory Cytokines and mTOR/AKT Signaling Pathway

**Figure 4:**
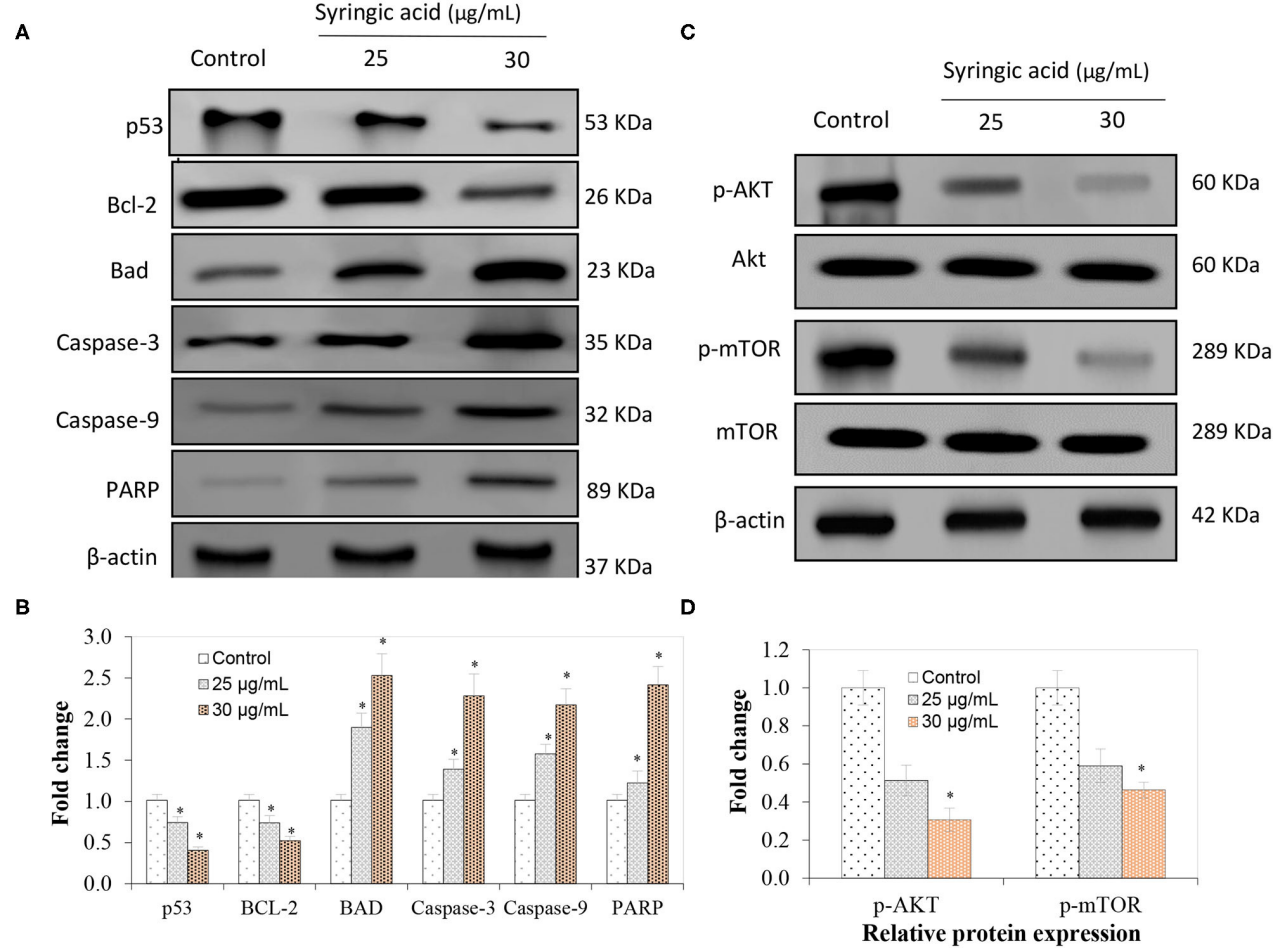
Regulatory effects of syringic acid at 25 and 30 μg/mL concentration on apoptotic markers of gastric cancer cells and expression pattern of **(A)** p53, BAD, BCL-2, caspase 3, caspase 9, PARP; **(B)** the fold changes induced; **(C)** mRNA expression of inflammatory markers; **(D)** relative protein expression of p-AKT and p-mTOR as quantified by Western blotting. Values are the mean ± standard deviation of 3 independent experiments, and statistical differences were determined at *P* < 0.05 level.

**Figure 5:**
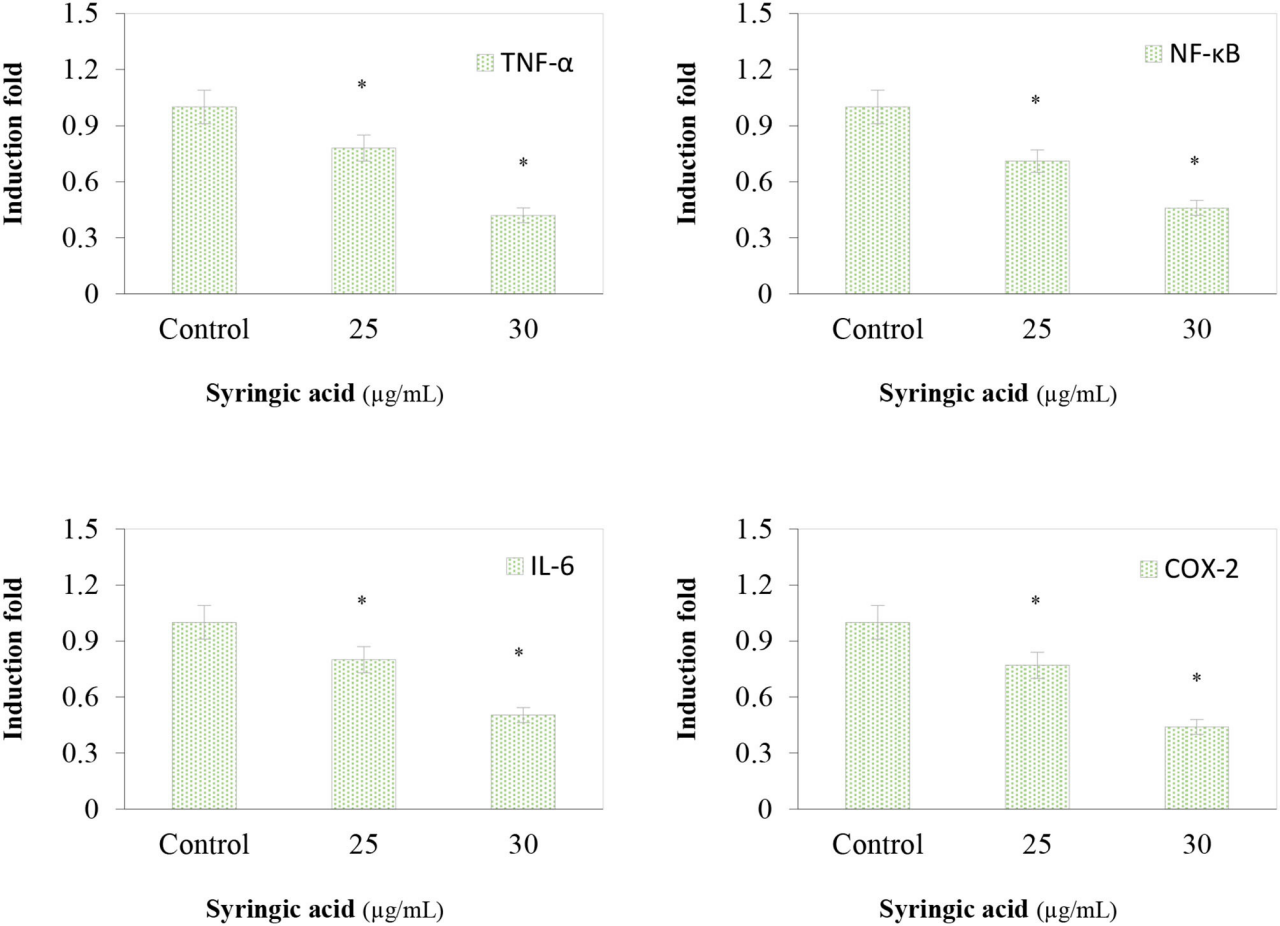
Effects of syringic acid (25 and 30 μg/mL) on inflammatory markers of TNF-α, NF-κB, IL-6 and COX-2 in gastric cancer cells. Values are the mean ± standard deviation of 3 independent experiments and statistical differences was determined at *p*< 0.05 level.

The immunoblotting analysis showed syringic acid at 25 and 30 μg/mL notably increased cleaved caspase-3, caspase-9, BAD and PARP whereas p53 and BCL-2 decreased (Figures 4A,B). The regulation of the mTOR/AKT signaling mechanism was also assessed to validate the effect of syringic acid on cells. Therefore, the occurrence of p-AKT and p-mTOR were assessed in syringic acid-treated cells, and results showed 30 μg/mL syringic acid effectively reduced the p-AKT and p-mTOR (Figures 4C,D) in which syringic acid down-regulated p-AKT and p-mTOR expression pattern. Other changes were also identified in the levels of AKT and mTOR (Figures 4C,D), indicating that phosphorylation of AKT and mTOR were downregulated by syringic acid. Findings, therefore, suggested syringic acid promoted apoptosis in cells by suppressing the activity of the mTOR/AKT. Suppressed expression of IL-6, NF-κB, TNF-α, and COX-2 in syringic acid-treated cells showed a more than 2-fold decrease at 30 μg/mL syringic acid concentration (Figure 5).

## Discussion

Syringic acid can induce apoptosis in various cancer cells (11). We recently reported syringic acid exhibited cytotoxicity properties toward oral squamous cell carcinoma SCC131 (14). Syringic acid, in the present study, suppressed the growth of gastric cancer cells in a dose-dependent manner and the morphological changes in syringic acid-treated cells demonstrated increased number of dead cells. Syringic acid can induce oxidative stress and apoptosis due to nuclear fragmentation and DNA damage (25, 26), therefore resulted in the appearance of late apoptotic mediators as detected by AO/EB, PI and DAPI techniques in our study as well.

Early apoptotic cells were observed at 25 μg/mL syringic acid (bright green nuclei) whereas late apoptotic cells were found to be at 30 μg/mL syringic acid concentration (orange nuclei). We therefore suggested syringic acid significantly induced apoptosis in doses between 25–30 μg/mL. Our findings also suggest syringic acid induced apoptosis via a mitochondrial-dependent pathway. The mitochondrial membrane potential (Δ*ψ*_m_) decreases with the leakage of electrons during the malignant cancer stage, in contrast, the depolarization of Δ*ψ*_m_ can be applied for inducing cancer cell death (27). The status of antioxidant enzyme in gastric cancer cells, in the present work, indicated an accumulation of ROS in syringic acid-treated cells. Our data also demonstrated syringic acid suppressed the release of pro-inflammatory cytokines (NF-κB, IL-6, COX-2, and TNF-α).

Syringic acid can inhibit cell proliferation via mitochondrial-mediated apoptotic pathways such as p53, cyt-C, BCL-2, Apaf-1, caspase-3, and caspase-9 proteins (28). Numerous anti-apoptotic proteins may be regulated such that cytochrome-c is leaked from mitochondria to the cytoplasm (27, 29) and contributes to the regulation of caspases (27). Our data indicated syringic acid selectively developed apoptosis in a dose-dependent manner via enhanced regulation of caspase-3, caspase-9 and PARP, whereas decreasing p53 and BCL-2 expression levels. The multi-stage development of gastric cancer is still not well understood. The mutation induction, regulation of oncogenes, and loss of tumor suppressor gene alteration are due to chromosome instability (30). The phosphorylated mTOR (p-mTOR) is a prognostic marker which occurs in the downstream processing of the PI3K/AKT/mTOR pathway in gastric cancer (31). The mTOR/AKT signaling plays a pivotal role in programming cell death and our data suggested syringic acid regulated the mTOR signaling pathway as the regulation of mTOR inhibited AKT.

## Conclusion

Our study showed how syringic acid induced apoptosis by enhancing pro-apoptotic mediators and inhibiting anti-apoptotic protein expressions. The introduction of syringic acid to gastric cancer cells mainly inhibited the development of inflammatory mediators via regulation of the AKT/mTOR signaling pathway. Understanding the mode of action of phytochemicals can provide the rationale for a combinatory therapeutic strategy to battle cancer more effectively.

Limitations of the current study are the variety of histological differences in gastric cancer and the physiological properties of the stomach and the metabolic reactions in the gastrointestinal tract which may influence the bioactivity of syringic acid.

Future study can be focused on oral administration of syringic acid in pre-clinical/clinical models and exploring how microbiota can be affected. Genetics, epigenetics, metabolomics, and gut-microbiota investigations can advance our understanding how physiological functions and microbiota could alter a healthy, normal gastric mucosa to gastric cancer.

## Data Availability Statement

The original contributions presented in the study are included in the article/supplementary material, further inquiries can be directed to the corresponding author/s.

## Author Contributions

JP, PV, and MZ: methodology, formal analysis, and writing original draft. PV, AV, and MZ: conceptualization and review and editing. JP: funding acquisition. ZF and MZ: supervision. MZ: project administration and visualization. ZF: resources. AV: review. All authors contributed to the article and approved the submitted version.

## Funding

This study was funded by the National Natural Science Foundation of China (31801563), a Special Support Plan for High-Level Talents in Shaanxi Province (for JP), and the Foundation of Shaanxi Sanqin Scholars Innovation Team.

## Conflict of Interest

The authors declare that the research was conducted in the absence of any commercial or financial relationships that could be construed as a potential conflict of interest.

## Publisher's Note

All claims expressed in this article are solely those of the authors and do not necessarily represent those of their affiliated organizations, or those of the publisher, the editors and the reviewers. Any product that may be evaluated in this article, or claim that may be made by its manufacturer, is not guaranteed or endorsed by the publisher.
